# SkinSage XAI: An explainable deep learning solution for skin lesion diagnosis

**DOI:** 10.1002/hcs2.121

**Published:** 2024-11-28

**Authors:** Geetika Munjal, Paarth Bhardwaj, Vaibhav Bhargava, Shivendra Singh, Nimish Nagpal

**Affiliations:** ^1^ Amity School of Engineering and Technology Amity University Noida Noida Uttar Pradesh India

**Keywords:** deep learning, skin lesions, explainable artificial intelligence, HAM10000, GradCAM, LIME

## Abstract

**Background:**

Skin cancer poses a significant global health threat, with early detection being essential for successful treatment. While deep learning algorithms have greatly enhanced the categorization of skin lesions, the black‐box nature of many models limits interpretability, posing challenges for dermatologists.

**Methods:**

To address these limitations, SkinSage XAI utilizes advanced explainable artificial intelligence (XAI) techniques for skin lesion categorization. A data set of around 50,000 images from the Customized HAM10000, selected for diversity, serves as the foundation. The Inception v3 model is used for classification, supported by gradient‐weighted class activation mapping and local interpretable model‐agnostic explanations algorithms, which provide clear visual explanations for model outputs.

**Results:**

SkinSage XAI demonstrated high performance, accurately categorizing seven types of skin lesions—dermatofibroma, benign keratosis, melanocytic nevus, vascular lesion, actinic keratosis, basal cell carcinoma, and melanoma. It achieved an accuracy of 96%, with precision at 96.42%, recall at 96.28%, *f*_1_ score at 96.14%, and an area under the curve of 99.83%.

**Conclusions:**

SkinSage XAI represents a significant advancement in dermatology and artificial intelligence by bridging gaps in accuracy and explainability. The system provides transparent, accurate diagnoses, improving decision‐making for dermatologists and potentially enhancing patient outcomes.

## Graphical abstract

The research was meant to disentangle the difficulties of skin lesion diagnosis using a multimodal approach. Our suggested technique offers unparalleled levels of transparency and interpretability in skin lesion categorization and marks a substantial improvement in the area via methodical methodology and meticulous refining. The research focuses on proposing.

i. A straightforward and reliable technique for the categorization of a variety of skin lesions.
ii. Explore different deep learning (DL) models in the diagnosis of skin lesions and evaluate their performance and effectiveness.
iii. Enhanced DL model's interpretability and transparency in dermatology with the help of explainable artificial intelligence techniques.
iv. Helping DL models to be easily incorporated into clinical practice further supporting in skin cancer prevention, management, and improved patient outcome.

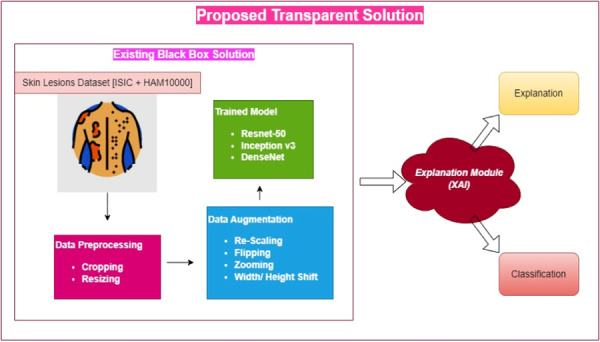

**Abbreviations**
AI: artificial intelligence
DL: deep learning
GradCAM: gradient‐weighted class activation mapping
LIME: local interpretable model‐agnostic explanations
XAI: explainable artificial intelligence

## 1 INTRODUCTION

The rising incidence of skin cancer, its high rates of morbidity and death, and its substantial health concerns make it a major global public health concern [1]. The severity of the issue is shown by recent epidemiological statistics that show a consistent increase in the incidence of skin cancer cases worldwide, which comes from reputable organizations like the World Health Organization and the American Cancer Society [2]. Many thousands of recent reports of melanoma, the most deadly kind of Skin Cancer, are diagnosed each year in the United States alone, but nonmelanoma skin cancer (NMSC) also indirectly targets many humans monthly and yearly [3, 4]. In the last several decades, there has been a rise in the prevalence of skin lesions and skin cancer cases, including melanoma and nonmelanoma. Globally, there are about 132,000 cases of melanoma and 2–3 million cases of NMSC annually. In the United States, one in five people will get skin cancer in their lifetime, and one in three cancer diagnoses is related to skin cancer, according to statistics from the Skin Cancer Foundation [5].

Skin cancer has heavy implications on social factors as shown; these include high costs of health care, a decrease in productivity, and a lower standard of living [6, 7]. The most dangerous reason for Skin Cancer is radiation from artificial sources that bring genetic alterations in the form of unchecked cell division and the development of cancerous growths in skin cells. Melanoma, squamous cell carcinoma, and basal cell carcinoma (bcc) are among the several conditions that fall under the umbrella term “skin cancer.” The etiology, clinical manifestation, and prognostic variables of each of these disorders are distinct. Despite being a less frequent kind of skin cancer than other types, melanoma is quite aggressive and has the ability to spread, therefore early identification and treatment are critical for the best results [7].

Skin lesions, a broad category of abnormalities related to dermatology, are important markers for underlying pathological processes and are essential for the diagnosis and treatment of skin cancer [8, 9]. These lesions may appear as benign moles or as malignant tumors; each variety has its own distinct morphological traits as well as histological characteristics. Different kinds of Skin Lesions existed—melanoma (mel), dermatofibroma (df), actinic keratosis (akiec), bcc, benign keratosis (bkl), melanocytic nevus (nv), and vascular lesion (vasc) [10, 11, 12, 13]. Skin cancer and other different skin diseases have already been traditionally detected by dermatologists with the help of dermoscopy, histological testing, and inspecting them by their eyes. Various different approaches have depicted efficacy and results; but, their inherited subjectivity, hard‐working nature, and susceptibility to interobserver variability could indirectly pose hardships in conquering consistent and believable diagnoses that can actually cure disease [14]. On the other side, because automated and incomplete subjects for analysis of skin lesions, live innovations in artificial intelligence (AI) and machine learning (ML), in particular, deep learning (DL) algorithms, have completely changed the field of dermatological diagnostics [15, 16, 17]. Large databases and annotated Skin Lesions pictures are already used to train DL models, which show expressive and outstanding accuracy in correctly identifying skin lesions and distinguishing between benign and malignant lesions thus improving physician's diagnosable precision.

Convolutional neural networks (CNNs)‐based DLs have shown high‐efficiency dermatoscopic image analysis for the diagnosing of malignant skin lesions, highlighting the tremendous influence of these technological advancements [18, 19, 20]. These techniques can provide medical practitioners with a potent tool that can learn from intricate patterns and features from large repositories of annotated images further navigating the complexities of lesion identification and classification [21, 22, 23]. The inherent complexity and opacity of DL models have raised concerns about their interpretability, which is crucial for gaining the trust and acceptance of health care professionals and patients. Even while DL‐based treatments in dermatology have enormous potential, there are still a number of obstacles standing in the way of their broad use and clinical integration.

DL models have impressive performance but their opaqueness in decision‐making processes is a major barrier to their acceptance in clinical practice integration [24, 25]. DL models, which are often characterized by their black‐box nature, are among important subjects of tension and concerns. These models are now able to depict outstanding accuracy in lesion classification tasks, but doctors are unable to properly decipher and trust the DL model predictions due to the black‐box nature [26]. Hence it becomes critical to explain the reasoning behind DL models' conclusions when they misclassify cases or provide unexpected results to ensure clinical usefulness and user acceptability [27]. Explainable DL techniques are thus highly needed, since they not only provide precise predictions but also offer valuable insights along with providing precise predictions.

Explainable artificial intelligence (XAI) approaches have emerged that facilitate clinician trust and cooperation by dissecting the underlying mechanics of DL models and explaining the reasoning behind their predictions [28, 29]. XAI is equipping medical professionals with needed and valuable tools along with insights to traverse the complex terrain of skin cancer detection confidently and precisely [30, 31].

This paper suggested framework “SkinSage XAI” that has incorporated the Inception v3 model because of its diagnostic precision and efficiency. The framework surpasses the constraints of conventional diagnostic methods by using the complementary powers of DL and XAI. Such system may enable dermatologists to fully use AI‐driven diagnostics without sacrificing accessibility or usability [32, 33, 34].

The frame provides the following features:

(1) A straightforward and reliable technique for the categorization of a variety of skin lesions.
(2) Explore different DL models in the diagnosis of skin lesions and evaluate their performance and effectiveness.
(3) Enhanced DL model's interpretability and transparency with the help of XAI techniques.
(4) Helping DL models to be easily incorporated into clinical practice further supports skin cancer prevention, management and improved patient outcomes.

The research is divided into four large segments. The first part is a thorough but crisp “*Literature Review*,” which majorly explains and routes through the body of research on skin lesion identification and classification for a variety of dermatological diseases. The methodology is based on the review, which automatically aligns with the various important procedures and viewpoints. Section 3 then goes into the methods and tools used, which include image labeling, augmentation strategies, data set description, CNN architectures that are used, and the SkinSage XAI framework that is specifically designed to improve skin lesion classification accuracy. The study also examines the well‐known XAI methodology's local interpretable model‐agnostic explanations (LIME) and GradCAM that provide light on DL models' decision‐making processes and promote interpretability and confidence in the classification outcomes. Section 4 thoroughly describes the acquired findings, their analyses, and a comparative assessment against current methodologies in skin lesion categorization. Section 5 concludes the study, which offers a reflection on the results and highlights the efficacy of the framework in diagnosing different types of skin lesions.

Skin cancer detection and treatment have received increased attention in recent years because of the pressing need to find practical ways to counteract the crippling condition's escalating incidence and prevalence. This has led to the conduct of many research using major methods and equipment to try to comprehend the difficulties involved in the diagnosis and categorization of skin cancer [35]. A number of data sets have been analyzed in this regard such as HAM10000 and the PH2 data set. These data sets have served as the basis for analyzing and evaluating CNN techniques like as DenseNet‐201, ResNet‐50, Inception v3, and so forth [1, 36, 37].

Various studies have shown the performance and effectiveness of different DL models in the diagnosis of skin lesions. ResNet‐18 and Inception v3 architectures are generally used in investigating skin lesion categorization and obtained an accuracy of 94.47% over eight lesion types [38]. A remarkable 86% accuracy rate was obtained by combining the VGG16 and ResNet‐50 designs across seven different lesion classes. The authors showed how occlusion‐based XAI methods might improve the transparency of the DL models and draw attention to the crucial components that influence diagnostic predictions. An accuracy of 94.92% is achieved using DL [39] this work enabled further developments in computer‐aided diagnosis. Shahin et al. [11] examined the effectiveness of hybrid DL models in the Inception v3 and ResNet‐50 architectures with a validation accuracy of 89.9%. Salido and Ruiz Jr. [13] also employed CNNs to classify skin lesions, using the PH2 data set and achieved an accuracy of 93% across three lesion types [40]. Sherif et al. [14] achieved an astounding 96.67% accuracy the model has shown its effectiveness in differentiating between benign and malignant lesions. Chowdhury et al. [18] investigated the categorization of skin lesions with a customized CNN method to interpret complicated patterns in dermoscopic pictures that achieved an accuracy of 82.7% for seven lesion classes. Esteva et al. [19] used the receiver operating characteristic (ROC) curve (area under the curve [AUC]) as a performance indicator for the DL model for discriminating benign and malignant lesions. Wilcoxon's sign rank test is used as a performance evaluator providing insight into the fundamental workings of DL‐based dermatological diagnoses [41]. ResNet‐50 and VGG16, along with the XAI model, are used in GradCAM [42]; the system achieved an accuracy of 76.7% across eight lesion classes. XAI has helped in understanding the decision‐making processes of DL models in dermatology by clarifying the key characteristics that underlie diagnostic predictions.

Using the ResNet‐50 architecture, Sadeghi et al. [21] studied skin lesion classification and obtained an accuracy of 60.94% for four lesion classifications. By using content‐based image retrieval approaches, their work demonstrated how DL may be used to retrieve pertinent photos for diagnostic reasons. Xie et al. [22] used the ISIC 2017 and PH2 data sets to investigate skin lesion categorization using a modified deep CNN. Using XAI, their research produced an accuracy of 90.4% across three lesion classes. Xie et al. [22] contributed significantly to our understanding of the interpretability of DL models in dermatology by clarifying the discriminative characteristics that underlie diagnostic predictions. Using the ISIC 2017 data set, Yang et al. [24] looked at the classification of skin lesions using the ResNet‐50 architecture that achieved an accuracy of 83% across two lesion classes.

XAI model GradCAM and kernel SHAP are explored for the categorization of skin lesions [25] and it attained an accuracy of 85% across two lesion classes. Table 1 offers a summary of research in the area, emphasizing important studies and their individual contributions [43]. DL is for attaining high diagnostic accuracy, opening the door for AI‐powered diagnostics in dermatology; however, these approaches often lack openness, interpretability, and explainability for which XAI approaches are to be adopted. Physicians should be able to understand data and make educated judgments during clinical procedures, our current work SkinSage framework is an effort toward this solution [44].

**Table 1:** A summary of various researches related to skin lesions.

Literature study	Data set(s)	Method	Outcome (%)	No. of skin lesions classes	XAI approach
Li et al. [7]	ISIC 2018	VGG16 + ResNet‐50	Accuracy 86.00	7	Occlusion
Kassem et al. [10]	ISIC 2019	Deep CNN	Accuracy 94.92	8	−
Shahin et al. [11]	ISIC 2018	Inception v3 + ResNet 50	Validation accuracy 89.90	7	−
Salido and Ruiz Jr. [13]	PH2	CNN	Accuracy 93.00	3	−
Sherif et al. [14]	ISIC 2018	Deep CNN	Accuracy 96.67	2	−
Chowdhury et al. [18]	HAM10000	Custom CNN	Accuracy 82.70	7	CAM
Esteva et al. [19]	ISIC 2018	CNN	AUC 94.00	7	Backpropagation
Sadeghi et al. [21]	ISIC 2019	VGG16 + ResNet‐50	Accuracy 76.70	8	GradCAM
Sadeghi et al. [21]	1021 Images	ResNet‐50	Accuracy 60.94	4	CBIR
Xie et al. [22]	ISIC 2017	CNN	Wilcoxon's sign rank test	7	CAM
Xie et al. [22]	ISIC 2017, PH2	Modified version of deep CNN	Accuracy 90.40	3	CAM
Yang et al. [24]	ISIC 2017	ResNet‐50	Accuracy 83	2	CAM
Young et al. [25]	HAM10000	Inception	Accuracy 85	2	GradCAM, Kernal SHAP
Hosseinzadeh Kassani and Hosseinzadeh Kassani [28]	ISIC 2018	ResNet‐50	Accuracy 92.00	7	−
Ünver and Ayan [29]	PH2, ISBI 2017	YOLO, Grab Cut	Accuracy 93.39	3	−
Nigar et al. [36]	ISIC 2019	ResNet‐18 + Inception v3	Accuracy 94.47	8	LIME
Nunnari et al. [42]	ISIC 2019	VGG16 + ResNet‐50	Accuracy 76.70	8	GradCAM
Zunair and Ben Hamza [45]	ISIC 2016	VGG16	AUC 81.18	2	CAM

Abbreviations: AUC, area under the curve; CAM, class activation mapping; CBIR, content‐based image retrieval; CNN, convolutional neural network; GradCAM, gradient‐weighted class activation mapping; ISBI, International Symposium on Biomedical Imaging; ISIC, International Skin Imaging Collaboration; LIME, local interpretable model‐agnostic explanations; ResNet, Residual Network; SHAP, SHapley Additive exPlanations; VGG; Visual Geometry Group; XAI, explainable artificial intelligence; YOLO, You Only Look Once.

## 2 METHODS

**Figure 1:**
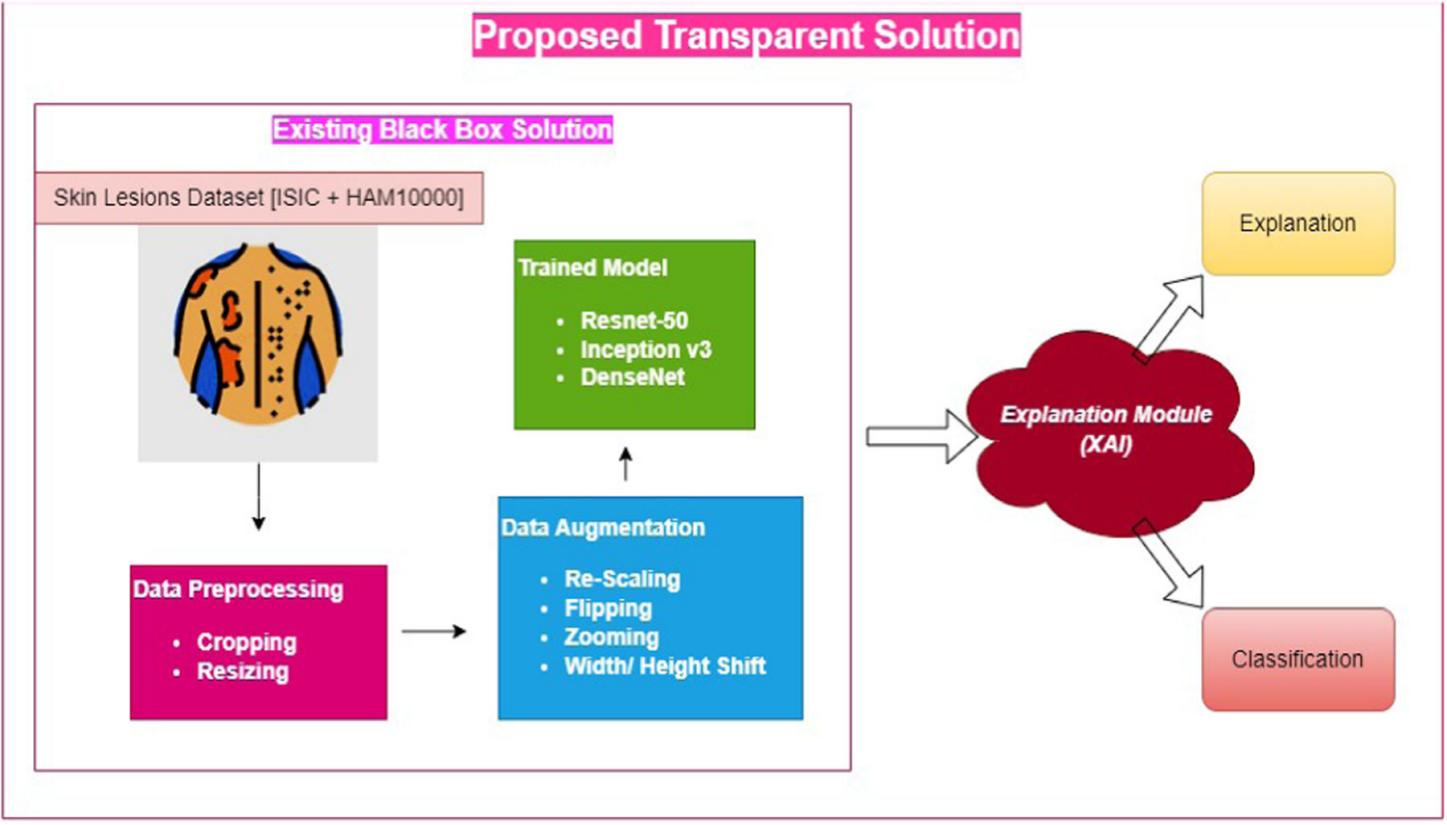
The Proposed Framework for SkinSage XAI. ISIC, International Skin Imaging Collaboration; ResNet, Residual Network; XAI, explainable artificial intelligence.

SkinSage XAI framework depicted in Figure 1 meant to disentangle the difficulties of skin lesion diagnosis using a multimodal approach. Our suggested technique offers unparalleled levels of transparency and interpretability in skin lesion categorization and marks a substantial improvement in the area via methodical technique and meticulous refining. The first task is data curation, which helps in model learning and provides stability and generalizability to the framework. HAM10000 is taken in the current study as it has an enormous amount of dermoscopic pictures. This data set has recognition in the ML and dermatology sectors, which have about 10,000 pictures in total, representing a variety of skin lesion types both benign and malignant.

After the data set is acquired, a thorough data preparation procedure is started, which is comprised of a variety of methods to maximize the accuracy and consistency of the input data. Preprocessing involves normalization and standardization of data which is essential to guarantee that the input characteristics have uniform scales and distributions. For optimized model building, homogeneity in data is encouraged, which also involves feature values rescaling using methods such as *Z* score standardization and Min–Max scaling. In the context of dermatoscopic images, feature engineering refers to the process of extracting pertinent attributes from the images, such as color, texture, shape, and boundary features, by using methods including edge detection, texture analysis, and morphological processes. These returned attributes serve as crucial inputs for DL systems, enabling accurate categorization and identification of significant features of skin lesions.

**Figure 2:**
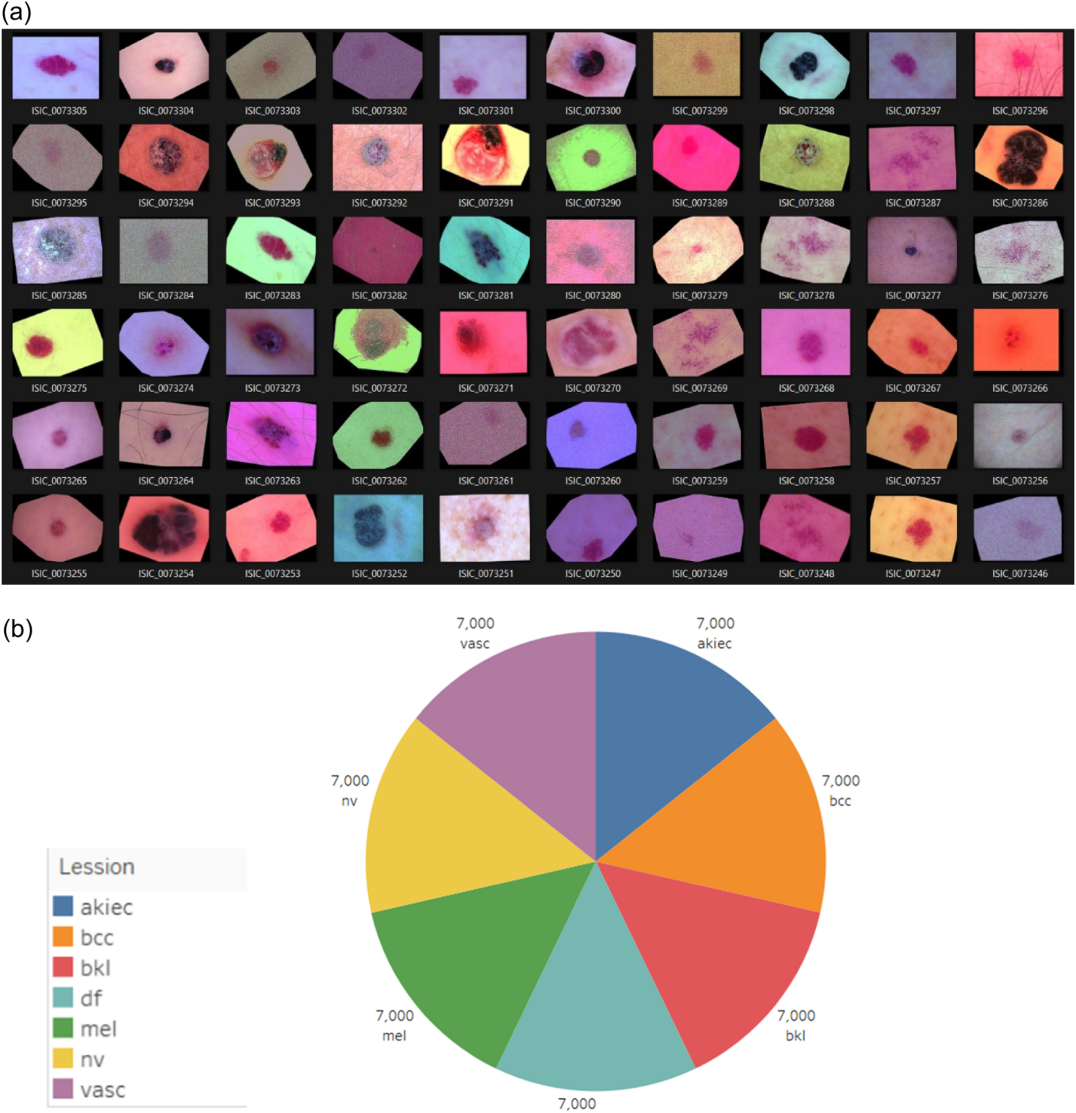
SkinSage XAI data set after applying data augmentation. (a) A snapshot of RGB images after data augmentation and (b) split of data set after data augmentation. akiec, actinic keratosis; bcc, basal cell carcinoma; bkl, benign keratosis; df, dermatofibroma; mel, melanoma; nv, melanocytic nevus; vasc, vascular lesion; XAI, explainable artificial intelligence.

For preprocessing, the data goes through a thorough augmentation process which includes rotation, translation, scaling, flipping, and color adjustments. Image rescaling is applied to normalize pixel values by scaling them within the range [0, 1]. Subsequently, image resizing ensures all images confirm to a consistent size suitable for the model's input dimensions. Augmentation techniques, including rotation, width and height shifts, and zoom, are employed to create diverse perspectives of the images, aiding in better learning. The rotation range introduces random rotations to the images, while width and height shift ranges enable horizontal and vertical translations, respectively. Zoom range applies random zooming in and out, and horizontal flip reverses images horizontally, further diversifying the data set. Additional augmentations like vertical flip and brightness range adjust the vertical orientation and brightness levels, respectively, to simulate various lighting conditions and viewpoints, thereby enhancing the model's resilience to real‐world variations. The model is trained separately on original and augmented data sets covering the whole area of lesions cases amongst these seven types. Figure 2 shows the data set after applying Data Augmentation: (a) a snapshot of RGB images after data augmentation and (b) split of data set after data augmentation among different classes. After obtaining the carefully curated data set, our attention turns to the hard training of DL models, which include the esteemed DenseNet‐201, ResNet‐50, and Inception v3 architectures. The above stated models are incarnated wisely to attain improved and peak performance along with dependability with the help of continuous cycles of training and validation. The most important assets and variables of the above models are wisely tuned (hyper) to get the final results. XAI models GradCAM and LIME are combined with the DL model, which provides priceless insights into the model's decision‐making methods and ways. LIME approximates the deep neural network model locally with an interpretable model to explain individual predictions, whereas GradCAM uses gradients of the target class in the form of a score flowing into the final convolutional layer to produce a coarse localization map highlighting important regions in the input image. The proposed methodology surpasses the hinderance of conventional black‐box methodologies by visualizing major parts and areas of interest as XAI can help in discovering which parts of an input image have the most impact on the forecasts generated by the model.

Different DL models are explored, including ResNet‐60, Inception v3, and DenseNet‐201; it is observed that Inception v3 is well adapted to the SkinSage XAI framework because of its memory and processing power. It enables faster system training and lower memory usage, making it more suitable for systems with limited resources. The Inception v3 integrates several regularization algorithms, such as batch normalization, to prevent overfitting and boost generalization efficiency. This is particularly critical for recognizing skin lesions since getting massive labeled data sets might be hard. In the overall process, the data set is divided into the train, validation, and test sets, followed by augmentation, as described in Section 2. After this step, training data become more diverse, and the model can learn better from different kinds of examples during the fitting process, thus being able to respond correctly even when presented with previously unseen cases.

Inception v3, whose lower layers are trained using the ImageNet data set, acts like a feature extractor where an input image is transformed into a hierarchical representation of learned features through transfer learning in the underlying model. Additional convolutional layers are used to enhance its learnt features effectively, that is, a 2D convolutional layer of size (3, 3) with 128 filters. Each filter learns different transformations over the original image patch during the filtering process; thus, spatial locality gets exploited while identifying such structures across feature maps generated after iteration. Further batch normalization layer standardizes the activations of the last convolutional layer, which minimizes the covariate displacement issue and training is accelerated by normalizing the input to each layer. The global average pooling layer creates a concise representation of the learnt features by combining spatial data from the whole feature map. This layer combines spatial data from the entire feature map, strengthen translation invariance, this pooling technique enhances the model's interpretability. Dropout layers are added after the GAP layer to make the model more general and prevent overfitting. During training, the dropout layer randomly deactivates certain neurons to help the network create multiple representations and reduce reliance on single units. This makes feature learning more robust and diverse which enables the model to generalize well with unseen data. Deep dense layers provide semantic abstraction as well as high‐level feature fusion. Two dense layers with 1024 and 256 units thick each are used in this method to capture complex interaction among features. The output layer employs the softmax activation function and helps in determining class probabilities based on acquired knowledge regarding various skin lesion types.

The models are trained separately with the original data set then the augmented data set will majorly cover the whole area of lesion cases amongst these seven types. The model is also fine‐tuned and has suitable parameters.

It is very important to understand how the Inception v3 and other DL models are able to make predictions, by using XAI methodologies, the gap between the intricate internal procedures of DL models and the level of explainability needed by humans to accept the results and trust the system gets enhanced. Transparency inclusion in XAI procedures helps to make the decision‐making processes clearer by providing timely insights into their internal procedures. With the aid of XAI, policymakers and developers may eliminate discriminatory trends and hidden biases in DL model predictions, enabling the creation of more egalitarian AI systems.

SkinSage XAI explores LIME and gradient‐weighted class activation mapping (GradCAM), which is an open, simple surrogate model that closely resembles the behavior of the complex black‐box model within a constrained region of interest. Both models build confidence and enhance interpretability in ML systems by illuminating the way in which certain input features impact model predictions. LIME processing includes segmenting the input image into superpixels, that is, groups of nearby pixels with similar color and spatial proximity, which affects the resolution of the region under study. LIME provides detailed, localized explanations by approximating the model's behavior in the neighborhood of specific predictions. This allows for comprehensive and nuanced analysis, particularly useful for identifying subtle factors influencing the model's decisions. The GradCAM evaluates the gradient of the expected class score of a skin image in relation to the final convolutional layer's activations, this gradient explains how different spatial positions of an image help in determining output class scores, based on changes in convolutional filters weights. GAP is employed on these computed gradients to produce a spatially averaged single value for each feature map, indicating its relative importance in predicting the expected class. Information about relative values is maintained between channels where higher values indicate a greater contribution of corresponding maps in the prediction of specific categories, whereas lower contributing attributes are given less weight compared to significant attributes while feature ranking.

Detailed explanations of the experimental design and the procedure for calculating the results for SkinSage XAI are drafted in this section along with hyperparameters selection of models. It also offers a fully explainable analysis of the framework's its results and outputs, both objectively and subjectively. To establish the superiority and major effectiveness of SkinSage XAI in skin lesion classification, the results are compared with other DL models. Pseudocode for SkinSage XAI is depicted as Algorithm 1.

**Table:** 

**Algorithm 1.** SkinSage XAI framework for categorizing skin lesion types.
**Input:** A set of pictures 200 × 200 pixels each scaled in size of skin lesions divided into training, validation, and testing sets in ratio 70:15:15; learning rate (*η*), number of epochs (*δ*), batch size (*λ*), number of photos per batch (*σ*); weights (*ω*) output by the CNN model. **Start** 1. Resized all images in the data set (HAM10000) on skin lesion to have equal dimensions within every class. 2. Use data augmentation techniques to equalize the data set size and improve the generalization ability of the DL models. 3. Extract features from the skin lesion data set using pretrained CNN models, which are DenseNet‐201, ResNet‐50 and Inception v3. 4. Change the top layers of CNN models for better classification of skin lesions: a. Add more layers with correctly labeled activation functions, for example, Swish or ReLU. b. Include some dropout layers to reduce overfitting. c. If there are multiple classes that need to be categorized, then use the softmax layer. 5. Assign default configurations to learning rate (*η*), number of epochs (*δ*), batch size (*λ*), and number of images per batch (*σ*) for CNN models. 6. Train each CNN model using the training data set: a. Initiate the task and run it for 150 rounds. b. Choose *σ*‐sized images from the training set as a mini‐batch. c. Predict the probability of each class using forward propagation. d. Compute loss by expected probability and actual label. e. Optimize selected optimizer Adam to update model weights with gradient backpropagation. f. Monitor accuracy and loss as training metrics. 7. Evaluate learned models with validation data set: a. Calculate validation loss and accuracy for assessing model performance. 8. Validation metrics based on the best performing model selection. 9. Utilize XAI explainability and analyze it using algorithms like LIME and GradCAM: a. To visually foreseen the areas of interest in the input pics and provide context for the model's predictions, use saliency maps and heatmaps. **End**

## 3 RESULTS AND DISCUSSION

A wider range of skin conditions and symptoms in the form of images are presented to models that help in improving model robustness. Subsequently, these data sets are split into training sets (validation set included); testing sets are created maintaining equal sample size per each type of lesion selected. By partitioning like this, it ensures unbiasedness while preventing overfitting, thereby enabling a more detailed investigation into model performance at the 70:15:15 ratio. With the aid of optimization strategies and well‐chosen hyperparameters, CNN models pick up a lot of knowledge during the training phase. DL models ResNet‐50, DenseNet‐201, Inception v3 have unique architectural components and learning capabilities, they are suitable for dealing with complexity and uncertainty associated with dermatological images where the Inception v3 have achieved best results. Higher level fully connected layers above the pretrained base of the Inception v3 model help in extracting everything else onto ImageNet data set.

All the experiments are done on the system with details depicted in Table 2, with the aid of optimization strategies and well‐chosen. A trade‐off between stability and convergence speed is minimized by using a learning rate of 0.001, while processing efficiency and model generalization are balanced by using a batch size of 16. The models are trained across more than 150 epochs, which allow them to continuously improve their representations of the underlying data and acquire discriminative qualities for the categorization of skin lesions. In the training phase, the Adam optimizer dynamically adjusts the learning rate to guarantee a seamless and effective convergence toward the ideal solution. The CNN models' decision‐making ability and procedures are provided by the system using XAI.

**Table 2:** Environmental setup for SkinSage XAI.

Resource	Details
CPU	Ryzen 5 5600H @3200 MHz
RAM	16 GB
GPU	GTX 1650
Language	Python
Platform	Jupyter Notebook

Abbreviations: CPU, central processing unit; GPU, graphics processing unit; GTX, giga texel shader extreme; RAM, random access memory.

**Figure 3:**
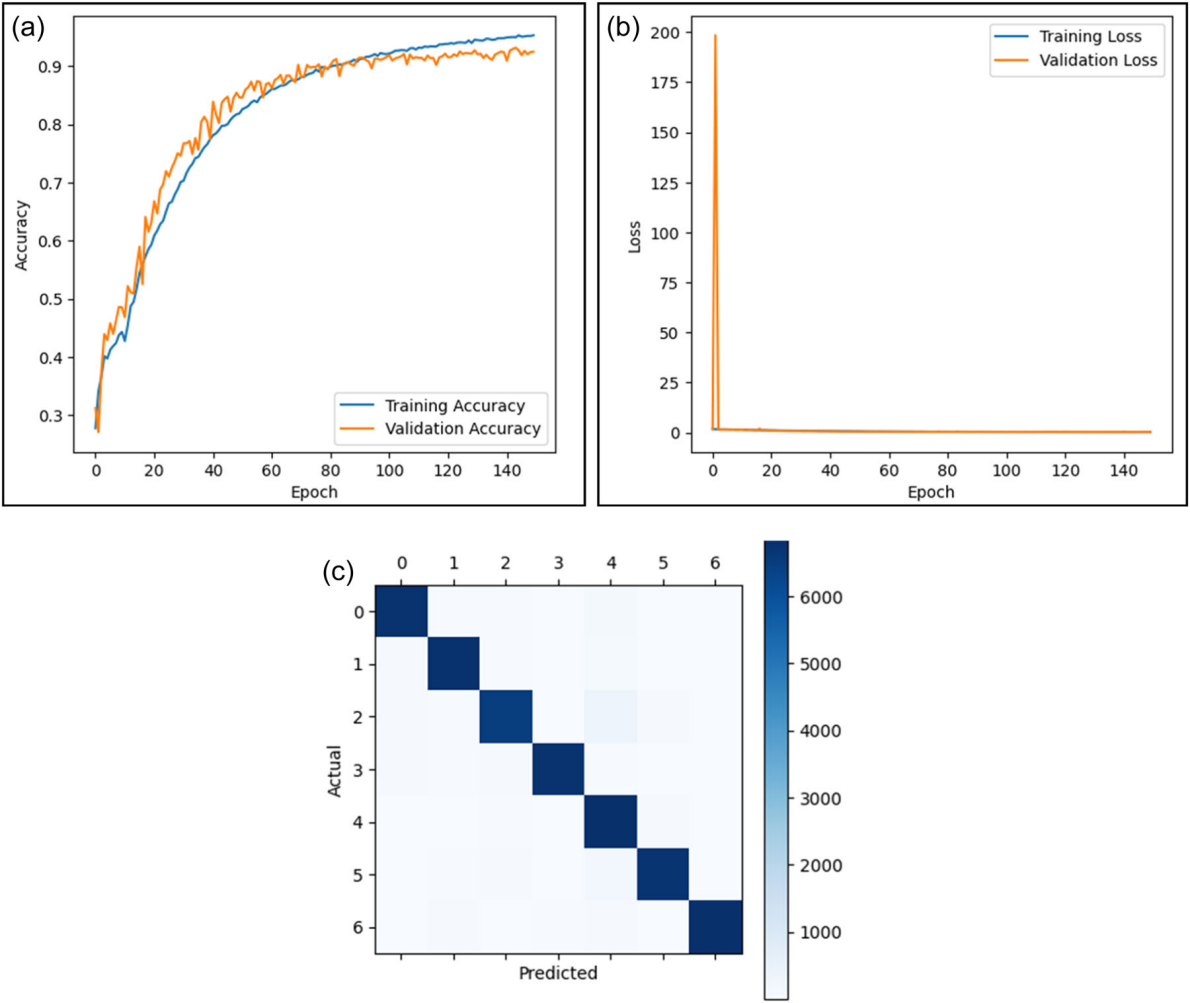
Results for the Inception v3 model with two graphs: one for accuracy and one for loss and with a confusion matrix. (a) Training and validation accuracy graph after a full train on 150 epochs, (b) training and validation loss graph after a full train on 150 epochs, and (c) confusion matrix for the predicted results in the Inception v3 model.

Figure 3 in displays the result of the Inception v3 where a training accuracy of 96% is a sign of its robustness and generalization capability. The model is able to classify even unforeseen images correctly and rapidly beyond what was taught to it as can be inferred from its near‐perfect accuracy on training data set. A model's validation accuracy shows how well it could predict unseen data; thus, giving us confidence that it will work when used in real‐life applications or situations where such forecasting is needed most. Additionally, good performances were noted for the Inception v3 models, which scored high levels concerning precision rates coupled with recall values while taking into consideration factors like accuracy, recall, *F*_1_ score etcetera inform of confusion matrix values. The model efficiently reduces false positives, with an average precision of 96.43%. The average recall of the model, which indicates that it can recognize a significant percentage of true positives, is 96.29%, indicating its sensitivity to identifying relevant events in the data set. Another thing worth mentioning here is that false positives/negatives were also considered during the calculation process thereby making an average *F*_1_ score of about 96.14%. On the basis of test accuracy alone, this makes the Inception v3 the best performing among other models integrated into the SkinSage XAI framework but not limited only by great validation power but also precision‐recall‐*F*_1_ measures. We can clearly observe an increase in accuracy along with a decrease in loss as epochs go by, showing this model has improved over time during training stages, thus indicating its usefulness for skin lesion classification tasks and suggesting possible application areas within dermatological diagnosis. Moreover, values of the Confusion Matrix, as in Figure 3c, are represented in Table 3 *where the class description is given in* Table 4.

**Table 3:** Confusion matrix values for the Inception v3.

Class	akiec	bcc	bkl	df	mel	nv	vasc
akiec	**6755**	42	48	23	126	4	2
bcc	70	**6769**	52	14	83	8	4
bkl	77	37	**6484**	14	329	56	3
df	77	28	70	**6761**	47	1	16
mel	22	15	38	1	**6849**	71	4
nv	5	30	72	1	177	**6714**	1
vasc	20	63	10	30	75	2	**6800**

*Note*: The bold values depict the number of samples correctly classified by the model.

Abbreviations: akiec, actinic keratosis; bcc, basal cell carcinoma; bkl, benign keratosis; df, dermatofibroma; mel, melanoma; nv, melanocytic nevus; vasc, vascular lesion.

**Table 4:** Class names versus skin lesions types.

Class 0	Class 1	Class 2	Class 3	Class 4	Class 5	Class 6
akiec	bcc	bkl	df	mel	nv	vasc

Abbreviations: akiec, actinic keratosis; bcc, basal cell carcinoma; bkl, benign keratosis; df, dermatofibroma; mel, melanoma; nv, melanocytic nevus; vasc, vascular lesion.

A high accuracy is referred to by the model performance indicator as properly detecting both positive (where lesions are present) and negative (where lesions are missing) occurrences of different kinds of skin lesions. However, it is not always sufficient in assessing how well the model has performed overall, but the number of correct predictions made by the model, that is, the percentage of true positive predictions out of all positive predictions, also called sensitivity. This is used in SkinSage XAI to evaluate how well the model reduces false positives with nonlesion regions classified as lesions. If a system has high accuracy and detects positive cases correctly, there is less likelihood of wrong diagnosis or unnecessary medical interventions, whereas lower accuracy can result in ineffective treatment and overdiagnosis which negatively affect patient care. *F*_1_ score depicted in Tables 5, 6, 7 combines recall and accuracy measures into one single value that offers a thorough assessment of the model accuracy by taking into account both false positives and erroneous results. To correctly detect skin lesions while reducing misclassifications, the proposed framework balances accuracy and recall, as shown Table 5, with a high *F*_1_ score.

**Table 5:** Classification Report for the Inception v3.

Skin lesion	Precision	Recall	*F*_1_ score	Support
akiec	0.96	0.96	0.96	7000
bcc	0.97	0.97	0.97	7000
bkl	0.96	0.93	0.94	7000
df	0.99	0.97	0.98	7000
mel	0.89	0.98	0.93	7000
nv	0.98	0.96	0.97	7000
vasc	1.00	0.97	0.98	7000

Abbreviations: akiec, actinic keratosis; bcc, basal cell carcinoma; bkl, benign keratosis; df, dermatofibroma; mel, melanoma; nv, melanocytic nevus; vasc, vascular lesion.

Recall and accuracy trade‐offs, however, must be carefully considered while maximizing the *F*_1_. For instance, classification reports are as follows: Table 5 contains for the Inception v3, Table 6 contains for ResNet, and Table 7 contains for DenseNet‐201.

(1) $$\mathrm{Accuracy}=\frac{\mathrm{TP}+\mathrm{TN}}{\mathrm{TP}+\mathrm{TN}+\mathrm{FP}+\mathrm{FN}},$$

(2) $$\mathrm{Precision}=\frac{\mathrm{TP}}{\mathrm{TP}+\mathrm{FP}},$$

(3) $$\mathrm{Recall}=\frac{\mathrm{TP}}{\mathrm{TP}+\mathrm{FN}},$$

(4) $$F_{1}\;\mathrm{score}=\frac{2\;\times \;\mathrm{Precision}\;\times \;\mathrm{Recall}}{\mathrm{Precision}+\mathrm{Recall}}.$$

**Table 6:** Classification Report for ResNet‐50.

Skin lesion	Precision	Recall	*F*_1_ score	Support
akiec	0.94	0.90	0.92	7000
bcc	0.94	0.89	0.91	7000
bkl	0.82	0.92	0.86	7000
df	0.98	0.90	0.94	7000
mel	0.80	0.94	0.87	7000
nv	0.97	0.87	0.92	7000
vasc	0.97	0.97	0.97	7000

Abbreviations: akiec, actinic keratosis; bcc, basal cell carcinoma; bkl, benign keratosis; df, dermatofibroma; mel, melanoma; nv, melanocytic nevus; vasc, vascular lesion.

**Table 7:** Classification report for DenseNet‐201.

Skin lesion	Precision	Recall	*F*_1_ score	Support
akiec	0.59	0.46	0.52	7000
bcc	0.64	0.65	0.65	7000
bkl	0.53	0.54	0.53	7000
df	0.61	0.70	0.65	7000
mel	0.65	0.52	0.58	7000
nv	0.64	0.94	0.76	7000
vasc	0.97	0.74	0.84	7000

Abbreviations: akiec, actinic keratosis; bcc, basal cell carcinoma; bkl, benign keratosis; df, dermatofibroma; mel, melanoma; nv, melanocytic nevus; vasc, vascular lesion.

In Equations (1)–(4) *true positives* (*TP*): The total number of positive cases that were accurately categorized.

*True negatives (TN)*: The quantity of properly categorized negative cases is known as “True Negatives.”

*False positives (FP)*: The quantity of negative cases that are mistakenly labeled as positive.

*False negatives (FN)*: The quantity of positive events that are mistakenly categorized as negative.

To sum up, Tables 5, 6, 7 depict that the SkinSage XAI framework provides satisfactory result in terms of accuracy, precision, and recall with all selected DL models which are the Inception v3, ResNet‐50, and DenseNet‐201 but the best performance is given by Inception v3.

**Figure 4:**
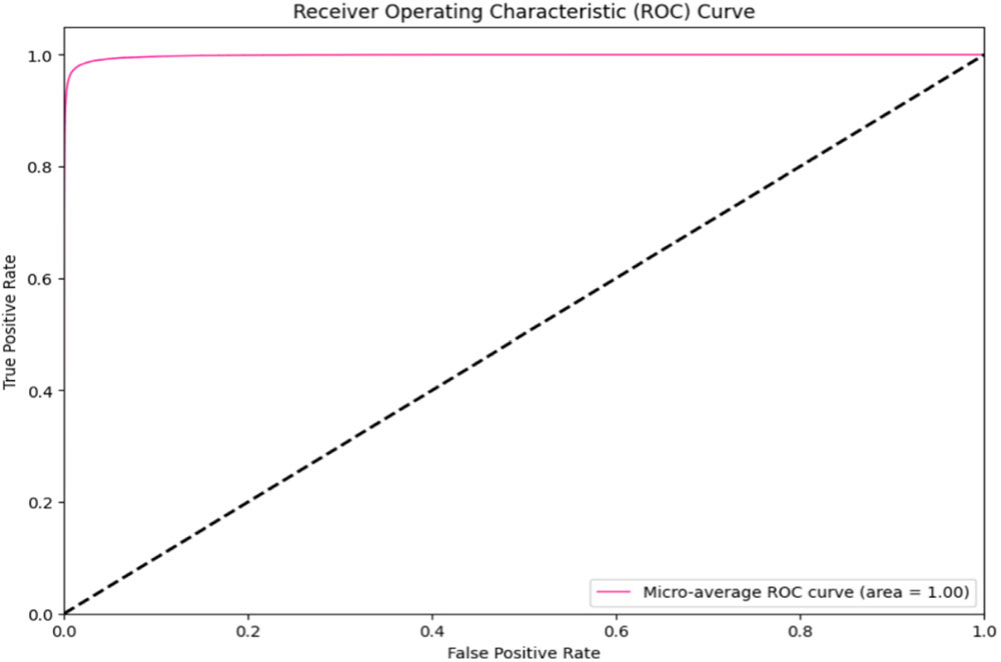
The Inception v3 model's ROC curve describes the model ability in distinguishing different kinds of skin lesions. This figure represents the overall ROC curve for the Inception v3 model. The AUC values vary among classes from 0.9960 to 0.9995. It can be seen class 0 (akiec) has an AUC of 0.9987 and class 1 (bcc) has an AUC of 0.9986. The model's overall efficacy in classifying skin lesions is further validated by the microaverage ROC curve, which has an AUC of 0.99. akiec, actinic keratosis; AUC, area under the curve; bcc, basal cell carcinoma; ROC, receiver operating characteristic.

Figure 4 provides the ROC analysis of Inception v3 model, each curve represents how a particular lesion category is classified. Importantly, high AUC values (0.9960–0.9995) for each class indicate the ability of the model to differentiate between positive and negative occurrences for different types of lesions. As in Table 3, class 0 (akiec) has an AUC of 0.9987, whilst class 1 (bcc) displays an AUC of 0.9986. Whereas the AUC values for classes 2 (bkl), 3 (df), 4 (mel), 5 (nv), and 6 (vasc) are, respectively, 0.9960, 0.9992, 0.9977, 0.9987, and 0.9995. This demonstrates the capacity of the model to categorize various skin diseases such as vascular lesions (vasc) and actinic keratoses (akiec). Also, the microaverage ROC curve with AUC equaling 0.99 reflects the overall reliability and effectiveness of the Inception v3 in the skin lesion classification task at large. When taken together, comprehensive ROC analysis clearly indicates that this model can accurately distinguish between different kinds of skin lesions.

The results generated by the DL model are not sufficient for medical practitioners to conclude a diagnosis. Thus, in the next step, XAI models are used to explain the results of the DL model. GradCAM and LIME XAI models are used to give these explanations.

GradCAM is able to make explanations with the help of heatmaps and, therefore, can explain the type of Skin Lesion predicted by the DL model. Heat maps are produced using activation maps from the last convolutional layer of the Inception v3 model, as shown in Figure 5. The purpose of these maps is to identify significant regions in an image that affect a model's prediction about a specific class label. GradCAM XAI model identifies those parts of an image which were essential for classification by placing heatmaps on top of original pictures. Heatmaps provide insights about parts of the image most influential toward projected class labels, thus enhancing interpretability and transparency for models. The GradCAM result shown next to the original image of Figure 5 helps identify the kind of skin lesion among the seven categories. The GradCAM output subsequently highlights the picture's visually important regions [46]. The heatmap illustrates the degree of activation across many image areas using a jet colormap and colorbar, which helps make sense of the model's predictions. by using this comprehensive visualization method, medical professionals will be able to perform more informed clinical assessments and comprehend the model's decision‐making process. LIME and GradCAM are both used in this study to interpret the model's predictions. LIME provides a more thorough and localized explanation, which is helpful for in‐depth examination, particularly for figuring out the precise elements influencing each prediction. GradCAM, on the other hand, offers a more comprehensive and understandable visual explanation; this is especially helpful for consumers who are looking for rapid and simple to understand insights. These approaches' capabilities complement one another, and when combined, they offer a strong foundation for model interpretability.

Figure 5Interpretations modeled by GradCAM. (a) Original image showcasing a bkl skin lesion. (b) GradCAM Result highlighting critical areas influencing the model's classification decision for the bkl skin lesion. (c) Heatmap with the “jet” colormap and colorbar illustrating the intensity levels of individual pixels, aiding in the analysis of regions influencing the model's decision‐making for the bkl skin lesion. (d) Original image presenting an nv skin lesion. (e) GradCAM Result indicating significant areas guiding the model's classification for the nv skin lesion. (f) Heatmap with the “jet” colormap and colorbar offering a visual representation of pixel intensity levels, aiding in the examination of regions affecting the model's decision‐making for the nv skin lesion. (g) Original image displaying a df skin lesion. (h) GradCAM Result highlighting crucial areas driving the model's classification for the df skin lesion. (i) Heatmap with the “jet” colormap and colorbar illustrating pixel intensity levels, facilitating the analysis of regions influencing the model's decision‐making for the df skin lesion. (j) Original image featuring a mel skin lesion. (k) GradCAM Result showcasing important areas guiding the model's classification for the mel skin lesion. (l) Heatmap with the “jet” colormap and colorbar providing a visual depiction of pixel intensity levels, aiding in the examination of regions affecting the model's decision‐making for the mel skin lesion. (m) Original image presenting a vasc skin lesion. (n) GradCAM Result indicating critical areas influencing the model's classification for the vasc skin lesion. (o) Heatmap with the “jet” colormap and colorbar illustrating pixel intensity levels, facilitating the analysis of regions affecting the model's decision‐making for the vasc skin lesion. (p) Original image displaying a bcc skin lesion. (q) GradCAM Result highlighting significant areas driving the model's classification for the bcc skin lesion. (r) Heatmap with the “jet” colormap and colorbar providing a visual representation of pixel intensity levels, aiding in the examination of regions influencing the model's decision‐making for the bcc skin lesion. (s) Original image showcasing an akiec skin lesion. (t) GradCAM Result indicating crucial areas influencing the model's classification for the akiec skin lesion. (u) Heatmap with the “jet” colormap and colorbar illustrating pixel intensity levels, facilitating the analysis of regions affecting the model's decision‐making for the akiec skin lesion. akiec, actinic keratosis; bcc, basal cell carcinoma; bkl, benign keratosis; df, dermatofibroma; GradCAM, gradient‐weighted class activation mapping; mel, melanoma; nv, melanocytic nevus; vasc, vascular lesion.

**Figure:**
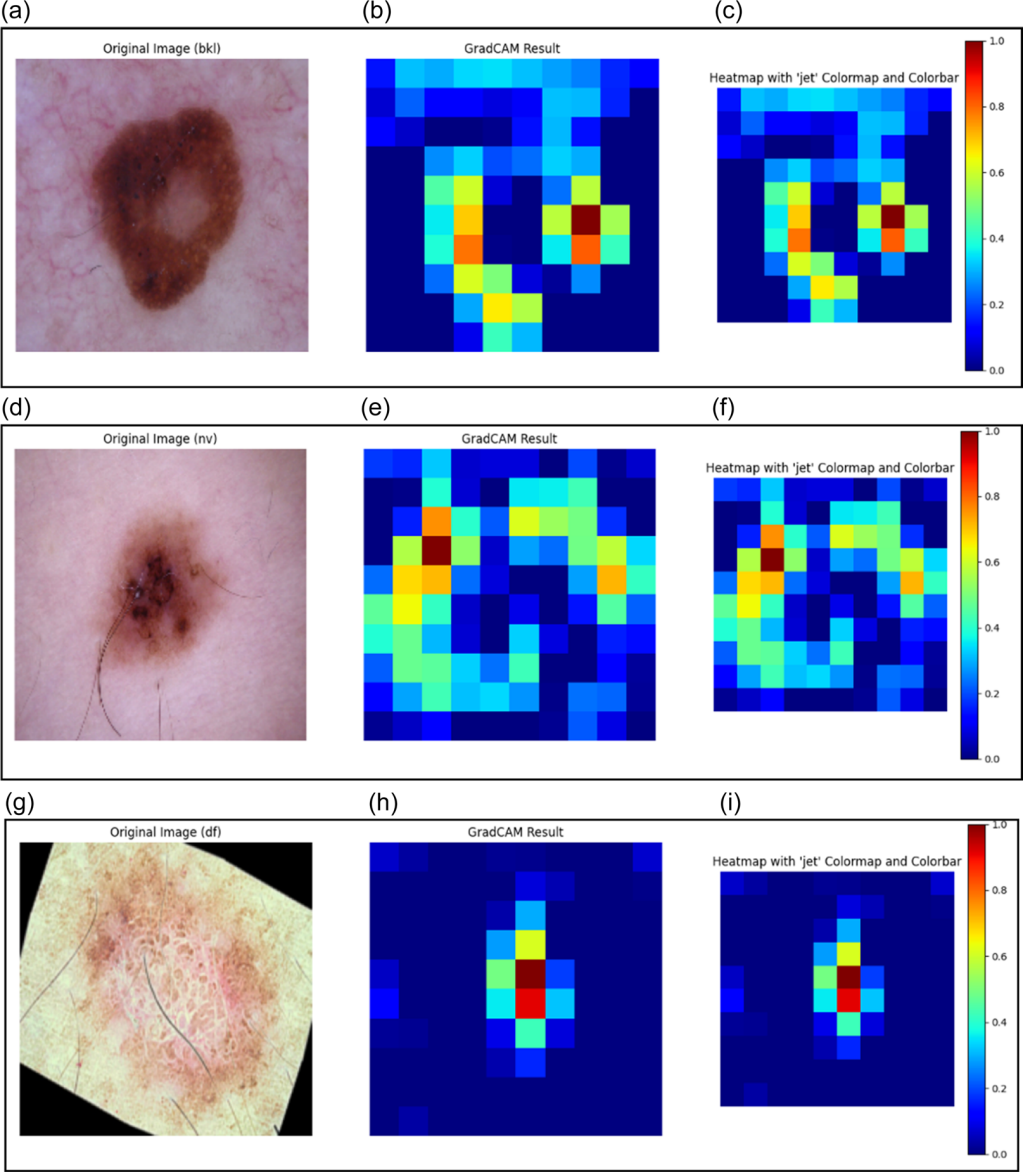

**Figure:**
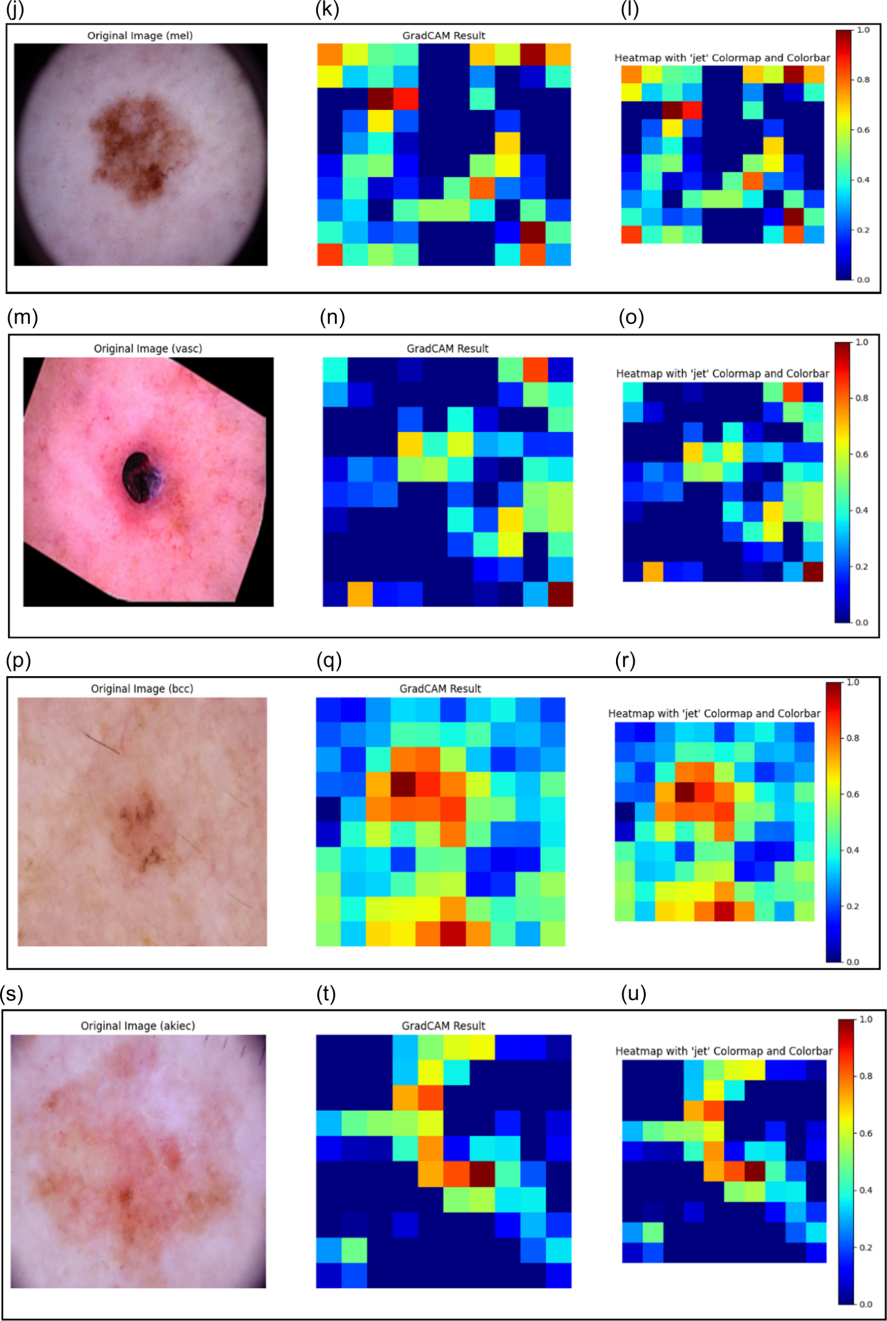

The Inception v3 model's decision‐making process is also explained using another LIME [46]. LIME is able to produce localized explanations for each prediction of different skin lesions. The capacity of LIME to recognize the crucial elements in a pic that could impact the model's prediction for a given class label. LIME works by segmenting the input picture into smaller areas called superpixels inside the framework of the SkinSage XAI.

As depicted in Figure 6, LIME has successfully predicted all the Skin Lesion Types, even in Figure 6c,e,n, LIME seems to not to perform well but it has still correctly predicted the Skin Lesion and it is just a Black‐box approximation tool which will explain the predicted the result of the model. In some cases, LIME has highlighted the major part on which it is able to explain that standalone results are predicted correctly. On the basis of the color and spatial closeness of pixels in the picture, these superpixels are generated. LIME helps identify certain parts that are most important to the model's decision‐making process by segmenting the picture into these coherent zones. The LIME interpretation process produces a number of different outputs. To give the following explanations a visual context, the original picture is first shown. The LIME picture with outline is then shown, with the key areas that LIME detected highlighted and outlined to emphasize their importance in affecting the model's forecast. The ultimate visualization shows a predicted lesion type of the model derived from the classification output and gives necessary background information about the class label assigned to the input image. Doctors can gain practical knowledge of the factors that influence model predictions through LIME in the SkinSage XAI platform, thus enabling them to have more trust and comprehension of AI‐based diagnosis.

Figure 6Interpretations modeled by LIME. (a) Original skin lesion photograph displaying features indicative of nv. (b) LIME interpretation highlights the regions with the most influence on the model's prediction for the nv lesion. (c) Predicted Skin Lesion Type: nv. (d) Original skin lesion photograph capturing characteristics indicative of a vascular lesion. (e) LIME interpretation revealing the regions with significant influence on the model's prediction for the vasc lesion. (f) Predicted Skin Lesion Type: vasc. (g) Original skin lesion image featuring characteristics suggestive of akiec. (h) LIME interpretation showcasing influential regions guiding the model's prediction for the akiec lesion. (i) Predicted Skin Lesion Type: akiec. (j) Original skin lesion photograph displaying features indicative of df. (k) LIME interpretation showcasing regions with significant influence on the model's prediction for the df lesion. (l) Predicted Skin Lesion Type: df. (m) Original skin lesion image exhibiting characteristics suggestive of a bcc. (n) LIME interpretation highlighting influential regions guiding the model's prediction for the bcc lesion. (o) Predicted Skin Lesion Type: bcc. (p) Original skin lesion photograph capturing features indicative of bkl. (q) LIME interpretation showcasing regions with significant influence on the model's prediction for the bkl lesion. (r) Predicted Skin Lesion Type: bkl. (s) Original skin lesion image featuring characteristics suggestive of MEL. (t) LIME interpretation highlighting influential regions guiding the model's prediction for the MEL lesion. (u) Predicted Skin Lesion Type: mel. akiec, actinic keratosis; bcc, basal cell carcinoma; bkl, benign keratosis; df, dermatofibroma; LIME, local interpretable model‐agnostic explanations; mel, melanoma; nv, melanocytic nevus; vasc, vascular lesion.

**Figure:**
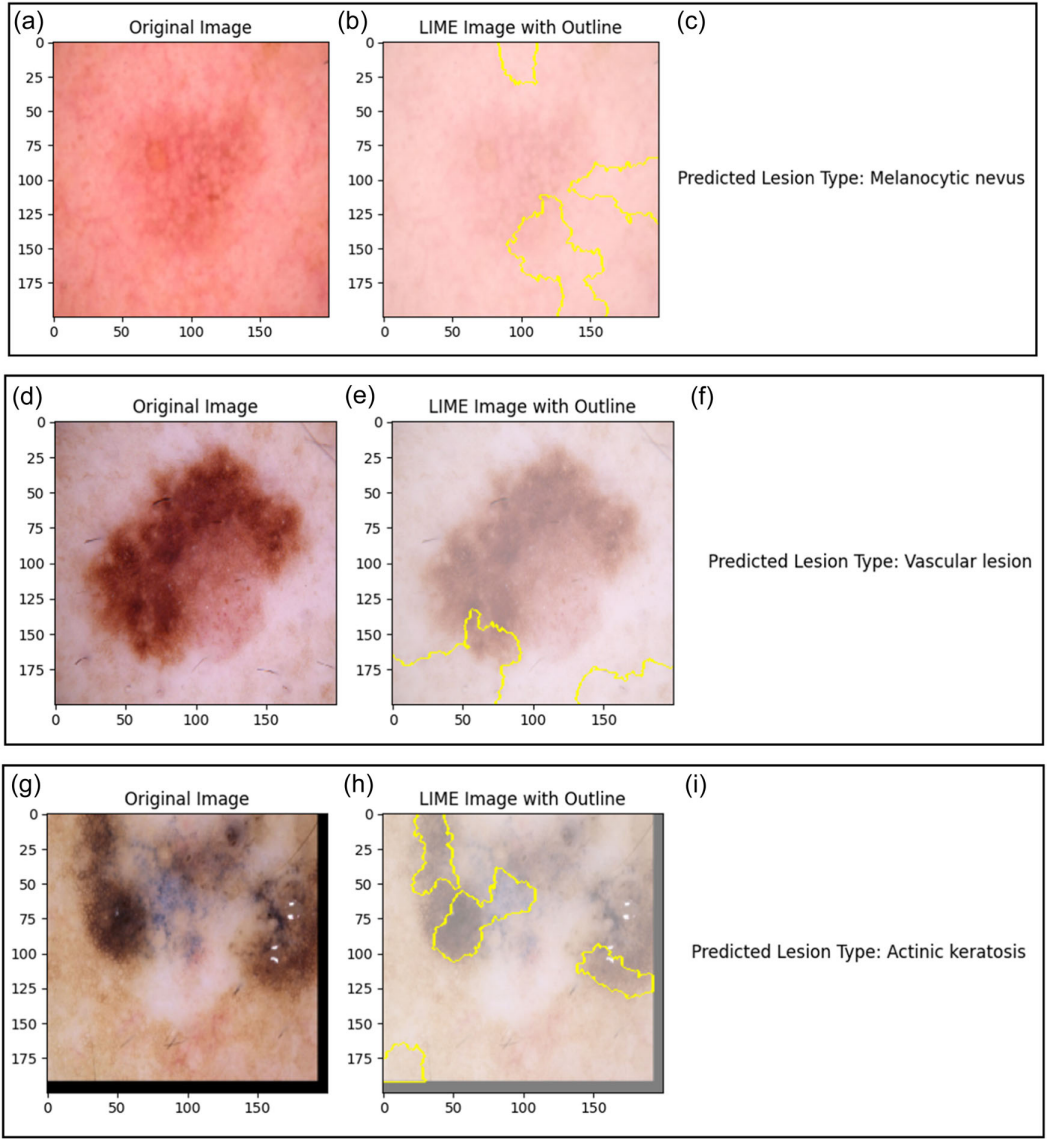

**Figure:**
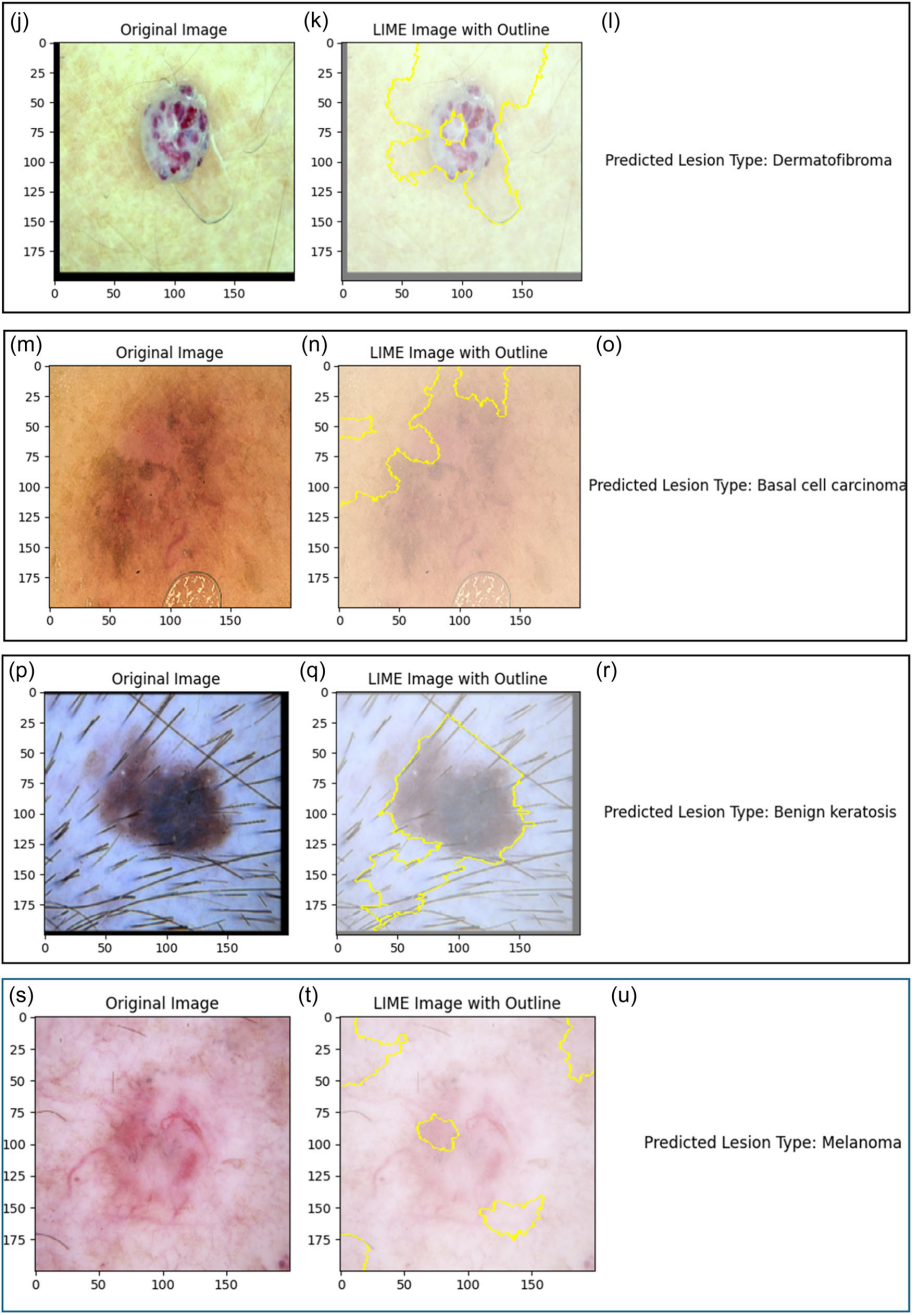

## 4 CONCLUSION AND FUTURE DIRECTIONS

To facilitate faster, transparent and dependable detection of skin lesions by dermatologists and other medical practitioners; this is a major breakthrough in the field of dermatology. With advanced DL models such as CNN, ResNet‐50, DenseNet‐201, and the Inception v3, among others; various types of skin cancers are studied and classified under SkinSage. Dermatologists may use a holistic approach, which will help them make better decisions on patient treatment, thereby leading to improved outcomes in patients' health. The framework presented provides clear visibility into how diagnoses were arrived at using the XAI model GradCAM and LIME. These models explained the decision‐making processes behind DL models. The assessment measures such as accuracy, precision, recall, and *F*_1_ score reveal that SkinSage performs very well; the Inception v3 model being the best‐performing model. The Inception v3 model has excellent classification ability with the highest validation accuracy of 93.41% and 96% as test accuracy. Such a system can reduce the time taken during lesion examination, hence making doctors concentrate more on challenging cases. Due to its simplicity, this system can be easily adopted, enabling practitioners to insights generated using AI with minimum effort. In addition, SkinSage facilitates communication and cooperation among dermatological professionals by offering a platform for data‐driven investigation and interpretation. By using massive and standard data sets along ML algorithms, the system ensures that it provides its support in uncovering new information regarding the prevalence, causes, and effects of dermatological problems. Resnet‐50 and DenseNet‐201 also performed well, achieving an accuracy above 85% in both cases, but were not up to the mark like the Inception v3 due to complexity and overfitting issues. Also, the Inception v3 multiscale feature extraction ability held an upper hand in predicting the results correctly with high confidence in LIME and GradCAM results. To understand the model's predictions, this study used both GradCAM and LIME, each of which has advantages. GradCAM delivers clear, visual insights that are simpler to understand quickly, while LIME offers comprehensive, localized explanations that are useful for in‐depth examination. When combined, these techniques enhance one another and offer a more thorough comprehension of the model's decision‐making procedure. When their qualities are combined, the interpretability is improved and a larger range of people may access it.

Enormous amounts of data can be processed together with advanced analytical tools provided by SkinSage‐XI, enabling researchers with an innovative diagnostic procedure as well as training future generations of dermatologists. Additionally, given its accessibility and interpretability, medical professionals can adopt AI‐driven diagnostic concepts as invaluable teaching and research aids. Use XAI methods along with cutting‐edge DL models, which offer insights into how diagnoses are made, thereby improving patient outcomes while also raising diagnostic accuracy levels at the same time.

The system SkinSage provides evidence‐based recommendations for treatment along with resources so that individuals can make informed choices about their skin health thereby enhancing health outcomes and life qualities regardless of where they live or what their income level is. In other words, this means that the inclusion of drug recommendation features in the application represents a significant step forward in personalized dermatological treatment and patient engagement. SkinSage could change how dermatologic therapies are administered around the world and improve people's lives universally.

## AUTHOR CONTRIBUTIONS

**Geetika Munjal**: Project administration (equal); supervision (equal); writing—review and editing (equal). **Paarth Bhardwaj**: Formal analysis (equal); validation (equal). **Vaibhav Bhargava**: Conceptualization (equal); investigation (equal). **Shivendra Singh**: Conceptualization (equal); resources (equal); visualization (equal). **Nimish Nagpal**: Data curation (equal); investigation (equal); software (equal).

## CONFLICT OF INTEREST STATEMENT

The authors declare no conflict of interest.

## ETHICS STATEMENT

Not applicable.

## INFORMED CONSENT

Not applicable.

## Data Availability

The data HAM10000 [1] is taken from the repository and publicly available data set.
